# Zoonotic Approach to *Streptococcus agalactiae*: Integrated Analysis of Sympatric Dairy Cattle and Human Isolates

**DOI:** 10.1155/ijm/5123333

**Published:** 2026-04-20

**Authors:** L. B. Hernandez, J. Cadona, C. Cacciato, G. Gerez, M. Banchiero, A. Bustamante, A. M. Sanso

**Affiliations:** ^1^ Laboratory of Immunochemistry and Biotechnology, Faculty of Veterinary Sciences-UNCPBA, CIVETAN (CONICET), Tandil, Argentina; ^2^ Laboratory of Clinical and Experimental Microbiology, Faculty of Veterinary Sciences-UNCPBA, CIVETAN (CONICET), Tandil, Argentina

**Keywords:** AMR genes, bovine mastitis, human colonisation and infection, MLST, *Streptococcus agalactiae*, virulence profile, zoonosis

## Abstract

*Streptococcus agalactiae* (Group B *Streptococcus*, GBS) is one of the most important pathogens causing bovine mastitis which also can colonise humans and cause severe diseases. To investigate the potential hazard of interspecific transmission, we selected 215 isolates (150 human + 65 bovine ones) obtained in a same period and geographical area. Comparative analysis of these sympatric isolates was carried out in relation to serotype, virulence and antimicrobial resistance (AMR) genes, and a selection of them, studied by MLST. Six serotypes were detected: Ia and III predominated in humans, III and II, in cattle. Virulence genes *cpsA, cylE* and *hylB* were detected in all isolates, *bac*, *hvgA, lmb*, PI‐1, PI‐2a and *scpB*, only in human isolates; *bca, rib*, *spb1* and PI‐2b, with variable frequencies between host isolates. Pilus typing detected PI‐1 and PI‐2a only in human isolates. All bovine isolates were PI‐2b. Clustering analysis showed 59 virulence profiles, without shared virulence profiles between human and bovine isolates. In relation to AMR‐encoding genes, *ermB, tetM, tetO* and *aphA3* were detected in both origins; *linB*, only in bovine isolates; *aad6*, only in human ones. Genes *tetO* and *aphA3* were significantly associated with bovine isolates; *tetM,* with human ones. Selected isolates were sequenced in order to assign CC and ST. Two human infective isolates showed ST10 (CC12) and ST17 (CC17), one colonising isolate, ST147 (CC17) and two new STs were detected in bovine genomes. The genetic characteristics among the GBS isolates demonstrate differences between the two populations, questioning the GBS zoonotic status.

## 1. Introduction

*Streptococcus agalactiae*, also known as Group B *Streptococcus* (GBS), is an important pathogen both in humans and a range of other animal species, including cattle. GBS was first described in 1887 as a bacterium that infects cattle udders, causing mastitis and significantly reducing milk production [1]. Since 1938, when three reports of fatal postpartum infection were published, GBS was frequently associated with a large variety of serious human infections, among which sepsis and meningitis in neonates [2]. In adults, GBS colonisation of the throat, gastrointestinal and genitourinary tracts is common, and the incidence of invasive GBS disease continues to increase worldwide [3].

Comparative analysis of *S. agalactiae* isolates from different origins can provide useful information to understand the potential interspecies transmission, colonising ability and evolution of this pathogen. While comparative analyses of contemporaneous, sympatric, human and bovine isolates are desirable [4], most of the published studies have evaluated isolates from one or other origin obtained in different regions or during different periods.

Several methods for identifying and characterising GBS have been described. These include capsular typing, typing of surface proteins and typing of pili that mediate interactions with host cells [5]. The capsular polysaccharide (CPS) is an antigenic determinant and a major virulence factor as it interferes with complement‐mediated killing [6]. According to its structure, ten serotypes (Ia‐Ib, II–IX) have been described [7]. CPS is presented in combination with different surface proteins including α‐C and ß‐C, Rib, Lmb, C5a peptidase, HylB and β‐haemolysin [8]. The frequency of the genes that encode them depends on the host and temporal/geographical origin of the studied strains. Three pilus variants, included in two pilus islands (PI), named PI‐1, PI‐2a and PI‐2b have been reported in GBS. All characterised GBS strains harboured at least one variant or a combination of the two PI [5]. Pili plays an important role in GBS colonisation and disease progression and, therefore, it is suspected that the type of pilus likely impacts on GBS invasion, adaptation and host specificity [9, 10]. Several studies highlight that GBS strains from different origins usually harbour different pili variants, while the PI‐2a is more common in human isolates and PI‐2b is more frequent in bovine isolates [10–13].

The emergence of GBS clones able to cause human infections has been associated with the acquisition of tetracycline resistance determinants, especially the *tetM* gene, a marker of success among human‐adapted GBS lineages. While acquisition of tetracycline resistance might have been essential for the initial emergence of GBS, acquisition of additional resistance markers, such as erythromycin and clindamycin resistance, might have been other important step for its evolution as a human pathogen [14].

Some authors have detected differences between GBS human and bovine subpopulations [15] while others, on the contrary, have not found a clear distinction between populations from both origins, postulating the lack of specificity according to the host [4, 16–18]. In addition, the occurrence of bidirectional interspecies transmission between cattle and people, zoonosis/anthroponosis, has been postulated [11, 17], with transmission pathways including direct contact and the consumption of milk. A hypothesis still debatable suggests that GBS jumped from animals to humans at a certain point in evolution, fixing and then specialising in the new host [14, 19, 20]. Particularly, certain investigations hypothesised that transmission to humans may occur during milking, drinking contaminated milk or through environmental contamination because GBS can survive in places other than the bovine udder in the dairy farms [9, 21].

The aim of our research was to compare dairy cattle and human *S. agalactiae* sympatric isolates in relation to genetic virulence and antimicrobial resistance (AMR) profiles, and sequence types (ST). Our interest was to know if they are differentiated into subpopulations and the potential hazard of interspecies transmission.

## 2. Methods

Two hundred and 15 *S. agalactiae* isolates (150 human and 65 bovine ones), which were obtained in a same time frame (2016–2022) and in the same geographical area, Pampean region of Argentina (centre and centre‐south province of Buenos Aires), were selected in order to compare them in relation to their origins. Human isolates, obtained from asymptomatic women with 35–37 weeks of pregnancy (123 colonising isolates) and symptomatic people (27 infective isolates), were proved by health institutions (three hospitals and three biochemical laboratories). On the other hand, bovine isolates were recovered from cows presenting clinical or subclinical mastitis in 10 dairy farms located in one of most important milk‐producing regions of Argentina, the Mary Sierras Cuenca, Pampean region of Buenos Aires province.

Most of the isolates had been previously characterised in relation to serotype, and virulence and antibiotic resistance genes [22, 23], meanwhile 12 isolates (A33, B68‐B78) were analysed in this study. To confirm the species of the isolates, previously identified using standard biochemical criteria, a region of the monocopy regulatory gene *dltR,* specific to *S. agalactiae*, was amplified by PCR. Serotypes were assigned according to Imperi et al. [24], and 13 virulence genes associated with adhesion and colonisation, invasion, tissue damage and/or immune evasion and six AMR genes were also investigated by PCR according to Hernandez [22, 23].

The statistical associations between serotypes, virulence/AMR genes and profiles and origin of isolates were analysed by 2 × 2 contingency tables, chi‐square test (*χ* ^2^) and Fisher exact test, with a confidence level of 95%, using the software Epi Info 7.1.5.2. Odds ratio (OR) values were determined. The OR expresses the likelihood of an event occurrence using probabilities, and a significant association occurs when the OR value is > 1 [25]. A clustering analysis based on virulence profiles (an UPGMA dendrogram) was constructed using BioNumerics, vs 6.6 (Applied Maths, Belgium).

In order to assign ST and clonal complexes (CC), eight isolates selected based on origin, serotype and virulence/AMR profiles were sequenced, A27, A33, PC97, B28, B63, B70, B77 and B49 which was previously analysed [26]*.* MLST determination was performed using the algorithm proposed by Jones et al. [27] in PubMLST web [28]. Then, the MLST database was used to match isolates from both origin in other countries obtained during the same period. Genetic relationships, an MLST‐based minimum spanning tree (MST) were visualised using the GrapeTree Layout algorithm [29].

## 3. Results

**FIGURE 1:**
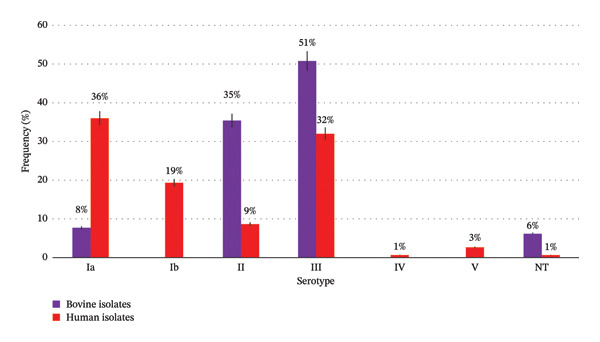
Serotype distribution in *S. agalactiae* bovine and human sympatric isolates.

A total of 215 *S. agalactiae* isolates (150 human isolates, and 65 bovine isolates) obtained between 2016 and 2022 from the Pampean region of Argentina were compared in relation to origin, serotypes and presence of genes involved in virulence and AMR. Six serotypes were detected. The serotypes’ distribution in all isolates into different origins is detailed in Figure 1. Serotypes III (38%), Ia (27%), II (17%) and Ib (13.5%) predominated among all isolates, followed by V (2%) and IV (0.5%), meanwhile two percent of the isolates were nontypeable (NT). In human isolates, serotypes Ia and III predominated, in cattle, serotypes III and II. Significant associations were found between serotype Ia and human isolates, as well as serotypes II, III and NT and bovine isolates (Table 1).

**TABLE 1:** Serotype‐origin association analysis in *S. agalactiae* bovine and human sympatric isolates.

Serotypes	No. (%) of isolates						Association between serotypes and origin	
	Total (*n* = 215)		Bovine (*n* = 65)		Human (*n* = 150)		OR (95% CI)	*p*
Ia	59	(27)	5	(8)	54	(36)	6.75	< 0.05
II	36	(17)	23	(35)	13	(9)	5.77	< 0.05
III	81	(38)	33	(51)	48	(32)	2.19	< 0.05
NT	5	(2)	4	(6)	1	(1)	9.77	< 0.05

*Note:* Significant association was found between serotype Ia and human isolates, and serotypes II, III and NT, and bovine ones.

**FIGURE 2:**
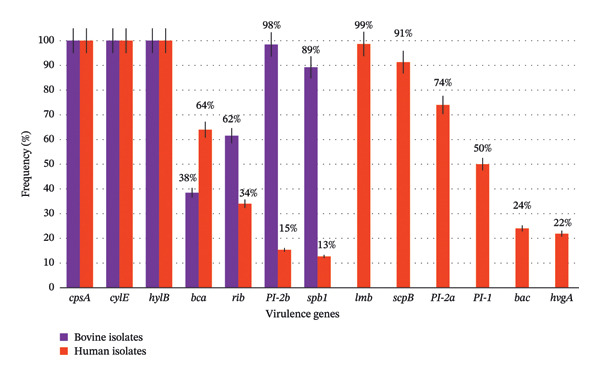
Distribution of virulence genes in *S. agalactiae* bovine and human sympatric isolates.

Virulence genes’ distribution is detailed in Figure 2. Genes *cpsA*, *cylE* and *hylB* were detected in all isolates, *bac, hvgA, lmb*, PI‐1, PI‐2a and *scpB,* only in human isolates. Comparative analysis showed significant positive associations between certain virulence genes and the host species, particularly *rib*, PI‐2b and *spb1*, with bovine isolates and *bca*, with human ones (Table 2). This would allow to infer that *rib* and *spb1* expression is linked to intramammary infection and that PI‐2b expression is linked to GBS’s ability to adhere to mammary tissue.

**TABLE 2:** Virulence gene–origin association analysis in *S. agalactiae* bovine and human sympatric isolates.

Virulence genes	No. (%) of isolates						Association between virulence genes and origin	
	Total		Bovine		Human		OR (95% CI)	*p*
*bca*	121	(56)	25	(38)	96	(64)	2.84	< 0.05
*rib*	91	(42)	40	(62)	51	(34)	3.10	< 0.05
*spb1*	77	(35)	58	(89)	19	(13)	57.13	< 0.05
*PI-2b*	87	(40)	64	(98)	23	(15)	353.39	< 0.05

*Note:* Significant association was found between *bca* and human isolates, and *rib*, PI‐2b and *spb1* and bovine ones (*p* < 0.05).

**FIGURE 3:**
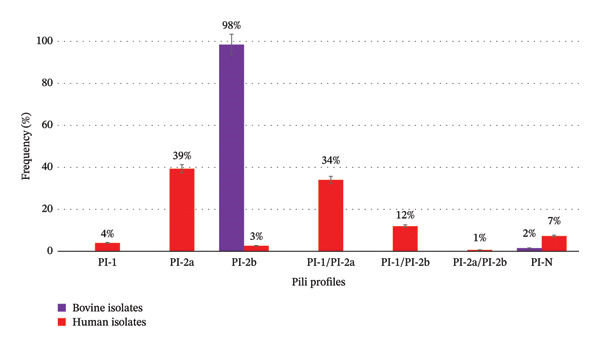
Pili profiles’ distribution in *S. agalactiae* bovine and human sympatric isolates. PI‐N: PI‐negative.

Bovine isolates showed the pilus profile PI‐2b, except one isolate which did not amplify any of the PI genes. In contrast, human isolates presented six PI profiles, and 7% were PI‐negative (Figure 3). Pilus typing detected PI‐1 and PI‐2a only in human isolates and PI‐2b, in both origins, with only 15% of the human isolates showing this gene. Of this total, 19 isolates were Serotype III, and 17 harboured *hvgA* (Table 3).

**TABLE 3:** Distribution of virulence profiles in PI‐2b‐positive human *S. agalactiae* isolates.

PI‐2b profile	Virulence profile	Serotype	*N*	Percentage of total PI‐2b‐positive human isolates (%)	Percentage of total human isolates (%)
PI‐1/PI‐2b	*hylB-cylE-cpsA-lmb*	II	1	4	0.7
	*hylB-cylE-cpsA-scpB-lmb-bca-bac*	Ia	2	9	1.3
	*hylB-cylE-cpsA-scpB-lmb-bca-bac-rib-spb1-hvgA*	III	1	4	0.7
	*hylB-cylE-cpsA-scpB-lmb-bca-rib*	III	1	4	0.7
	*hylB-cylE-cpsA-scpB-lmb-bca-rib-spb1*	III	1	4	0.7
	*hylB-cylE-cpsA-scpB-lmb-bca-rib-spb1-hvgA*	III	6	26	4
	*hylB-cylE-cpsA-scpB-lmb-rib-hvgA*	III	1	4	0.7
	*hylB-cylE-cpsA-scpB-lmb-rib-spb1-hvgA*	III	5	22	3

PI‐2a/PI‐2b	*hylB-cylE-cpsA-scpB-lmb-rib-hvgA*	III	1	4	0.7

PI‐2b	*hylB-cylE-cpsA-scpB-lmb-bca-rib-spb1-hvgA*	III	1	4	0.7
	*hylB-cylE-cpsA-scpB-lmb-rib*	II	1	4	0.7
	*hylB-cylE-cpsA-scpB-lmb-rib-hvgA*	III	1	4	0.7
	*hylB-cylE-cpsA-scpB-lmb-rib-spb1-hvgA*	III	1	4	0.7

			23	100	15

**FIGURE 4:**
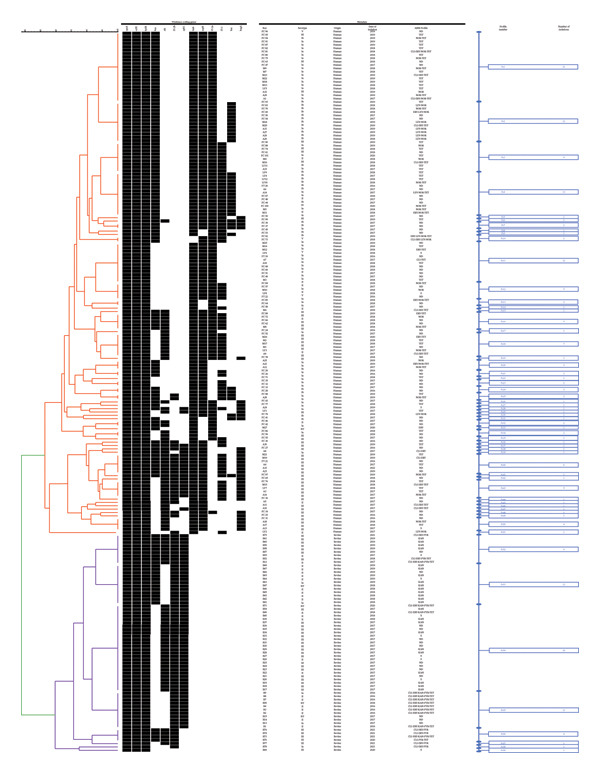
Clustering analysis of sympatric *S. agalactiae* human and bovine isolates. The presence (black) or absence (white) of virulence‐coding genes, the isolate name, serotype, isolation date and AMR profile of each isolate are shown. CLI: clindamycin; ERY: erythromycin; KAN: kanamycin; LEV: levofloxacin; NOR: norfloxacin; PEN: penicillin; PYR: pirlimycin; TET: tetracycline; S: susceptible to all the tested antimicrobials; ND: not determined. Each profile is named as “Ps” + a number. Red: human isolates; violet: bovine isolates.

Clustering analysis based on virulence genes grouped the 215 isolates into 59 virulence profiles, 8 profiles grouped the bovine isolates and 51, the human ones (Figure 4). Of the 8 bovine profiles, only 3 were unique and, of the 51 human profiles, 31 were unique. The most frequent profile in cattle isolates was *hylB-cylE-cpsA-rib-spb1*‐*PI-2b* (Ps53, 40%), and among the human isolates, *hylB-cylE-cpsA-scpB-lmb-bca-PI-2a* (Ps1, 14%). Bovine origin virulence profiles included between 2 and 7 genes, while those from human origin, between 5 and 12 genes. No shared virulence profiles between human and bovine isolates were found.

**FIGURE 5:**
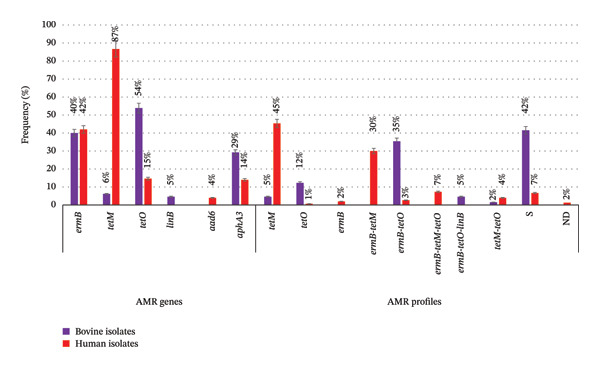
Distribution of genes encoding antimicrobial resistance and profiles. S: nondetection of analysed AMR genes; ND: not determined.

In relation to AMR‐encoding genes, *ermB, tetM, tetO* and *aphA3* were detected in both bovine and human isolates; *linB*, only in bovine ones, and *aad6*, only in human ones. The distribution of these genes varied between bovine and human isolates, with *tetO* and *aphA3* being significantly associated with bovine and, *tetM,* with human (Table 4). Isolates were grouped into 8 AMR genetic profiles, plus one negative for all analysed genes (S) and one without data (ND). Five profiles, including S profile, were shared between both origins (Table 4; Figure 5).

**TABLE 4:** Distribution of genetic antimicrobial resistance (AMR) in *S. agalactiae* isolates and association analysis between AMR and origin. (a) Association analysis between AMR genes and origin; (b) association analysis between AMR genetic profiles and origin.

**(a)**								
**AMR genes**	**No. (%) of isolates**						**Association between AMR and origin**	
	**Total**		**Bovine**		**Human**		**OR (95% CI)**	***p***

*aphA3*	40	(19)	19	(29)	21	(14)	2.54	< 0.05
*ermB*	89	(41)	26	(40)	63	(42)	1.11	> 0.05
*tetM*	134	(62)	4	(6)	130	(87)	99.13	< 0.05
*tetO*	57	(26)	35	(54)	22	(15)	6.78	< 0.05

**(b)**								
**AMR genetic profiles**	**No. (%) of isolates**						**Association between AMR and origin**	
	**Total**		**Bovine**		**Human**		**OR (95% CI)**	***p***

*tetM*	71	(33)	3	(5)	68	(45)	17.14	< 0.05
*tetO*	9	(4)	8	(12)	1	(1)	20.91	< 0.05
*ermB-tetO*	27	(13)	23	(35)	4	(3)	19.98	< 0.05
*tetM-tetO*	7	(3)	1	(2)	6	(4)	2.67	> 0.05
*S*	37	(17)	27	(42)	10	(7)	9.94	< 0.05

*Note:* Significant association was found between *tetO* and *aphA3* and bovine isolates, and *tetM* and human isolates. The presence of *ermB* was not associated with any origin. S: nondetection of analysed AMR genes. Significant association was found between *tetO, ermB-tetO* and S and bovine isolates and *tetM* and human isolates.

Seven of the eight sequenced isolates could be subtyped by MLST. Infective isolates A27 and A33 showed the ST10 (CC12) and ST17 (CC17), respectively, meanwhile the colonising isolate (P97) showed the ST147 (CC17). New STs were detected in two bovine isolates, ST1640 (B49) with a new allele for *sdhA* and ST2276 (B63), both not assigned to any CC. On the other hand, two bovine isolates (B70, B77) showed ST61 (CC17). The ST for B28 isolate could not be determined due to atypical *glcK* sequence (Table 5).

**TABLE 5:** Serotype, sequence‐type (ST) and clonal complex (CC) of the eight *s*equenced *S. agalactiae* isolates.

#	Isolate	Serotype	Origin	Virulence profile	AMR profile	ST (CC)	Reference
1	A27	Ib	Human infection	*cpsA-cylE-hylB-lmb-scpB-bca-bac-PI2a-*	*ermB-tetM-tetO*	ST10 (CC12)	This paper
2	A33	III	Human infection	*cpsA-cylE-hylB-lmb-scpB-bca-rib-spb1-PI1-PI2b-hvgA*	ND	ST17 (CC17)	This paper
3	P97	III	Human colonising	*cpsA-cylE-hylB-lmb-scpB-bca-bac-rib-spb1-PI1-PI2b-hvgA*	*ermB-tetM-tetO-aad6-aphA3*	ST147 (CC17)	This paper
4	B28	III	Bovine mastitis	*cpsA-cylE-hylB-PI2b-spb1-rib*	*aphA3*	ND	—
5	B49	II	Bovine mastitis	*cpsA-cylE-hylB-PI2b-spb1-rib*	*ermB-tetO-linB-aphA3*	ST1640 (NA)	[26]
6	B63	Ia	Bovine mastitis	*cpsA-cylE-hylB-PI2b-spb1-bca*	S	ST2276 (NA)	This paper
7	B70	III	Bovine mastitis	*cpsA-cylE-hylB-PI2b-rib-bca*	*ermB-tetO*	ST61 (CC17)	This paper
8	B77	III	Bovine mastitis	*cpsA-cylE-hylB-PI2b-rib*	*ermB-tetO*	ST61 (CC17)	This paper

*Note:* NA: CC not assigned.

Abbreviation: ND, not determined.

**FIGURE 6:**
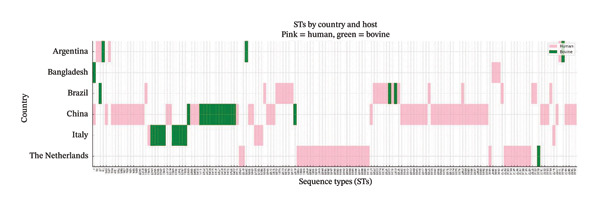
Presence of *S. agalactiae* sequence types (STs) in countries reporting isolates from human and bovine origin in the period 2016–2022.

Using the PubMLST database, it was possible to match 7631 bovine and human isolates in relation to the country. Of the total, 266 isolates (32%, cattle; 68%, humans) were obtained between the years 2016 and 2022, the period of this study, Argentine (7), Bangladesh (6), Brazil (33), China (122), Italy (11) and The Netherlands (87). A comparative analysis was performed to provide a synthesised visualisation of ST distribution by country and host, highlighting both unique and shared genetic lineages of *S. agalactiae* (Figure 6). In Argentina, in addition to the STs identified in this study, ST2271 was reported. Overall, the analysis revealed a wide genetic diversity of STs among countries. ST10, ST61, ST147, ST1640, ST2271 and ST2276 were unique to Argentina within this period. Particularly, ST4 and ST17 were present in one or other origin depending on the country; ST4 in Bangladeshi bovine isolates and Chinese human ones; ST17 in Argentinian human isolate and Brazilian bovine ones.

**FIGURE 7:**
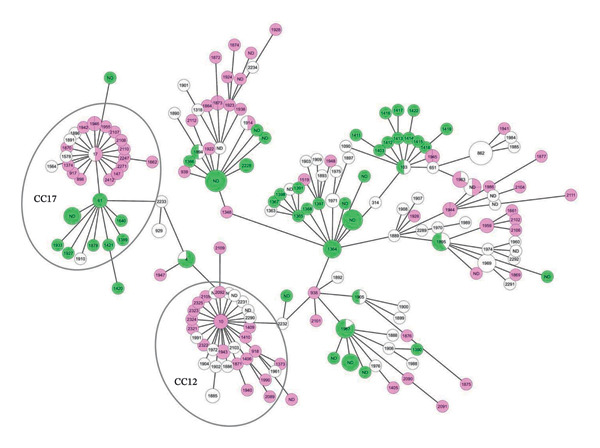
Minimum spanning tree of STs and CCs from 266 *S. agalactiae* isolates obtained between 2016 and 2022, using GrapeTree. Pink: human; green: bovine; white: not determined. Eleven isolates classified as “other animals” were excluded. The CC12 and CC17, clonal complexes detected in isolates analysed in this study, are highlighted.

In order to construct an MLST‐based MST, the data were analysed using the GrapeTree of PubMLST, based on the MSTreeV2 algorithm. The phylogenetic tree based on *S. agalactiae* isolates obtained between 2016 and 2022 from countries with recorded lineages of human and bovine origin was constructed with default parameters (Figure 7). Although these MLST data are exploratory due to the low number of sequenced isolates, in general, each ST was assigned to one or another host. CC12 was entirely composed of human‐derived isolates STs; in contrast, CC17 not only had a majority of STs identified in humans but also included bovine‐derived STs, suggesting shared ancestry. Also, several STs belonging to other CCs clustered closely despite differing origins, indicating potential epidemiological links. Notably, although ST4 and ST17 are shown in the figure with only a single origin, manual revision confirmed that both were detected from both human and bovine sources.

## 4. Discussion

In this study, *S. agalactiae* isolates obtained from humans (colonising and infective) and bovines (mastitis) from the Argentinean Pampa region over the same period (2016–2022) were compared taken into.

Of the six serotypes detected, serotypes Ia, II and III were common to isolates from both origins, as reported by Carra et al. [9], Pinto et al. [30] and Dogan et al. [31]. These authors detected also Ib, IV and V among isolates from both origins, whereas we only detected them in human isolates, as did Emaneini et al. [32]. On the other hand, Lyhs et al. [4] detected serotypes Ia, Ib and II in human and bovine origin, while VI was detected only in humans and V and VII, in cattle. Significant association was found between serotype Ia and human isolates, and between serotypes II and III, and cattle isolates.

*S. agalactiae* possess a variety of potential virulence factors. Regarding virulence‐encoding genes, *cpsA, cylE and hylB* were detected in all isolates, regardless of origin, while others, such as *bca, rib, spb1* and PI‐2b were detected in variable frequency in both origins and others, such as *bac, hvgA, lmb*, PI‐1, PI‐2a and *scpB* only in human isolates [22, 23]. Comparative analysis showed significant association between certain virulence genes and the origin of the isolates, *rib, PI-2b* and *spb1* with bovine and *bca,* with human. The *bca* gene was detected in 38% of bovine and 64% of human isolates, in contrast to that reported by Emaneini et al. [32], who did not detect this gene in bovine isolates. Other authors have reported the presence of *bca* in both origins [2, 33]. This gene encodes the Alpha C protein, which is involved in attachment to and entry into eukaryotic cells [34]. Sixty‐two percent and 34% of the bovine and human isolates, respectively, were *rib-positive*. The significant association detected between *rib* and bovine isolates is consistent with the high frequency detected by other authors, who have linked the presence of Rib to bovine mastitis development [32]. The *lmb* gene was detected only in human isolates, unlike some authors who found it also in cattle isolates [2, 33, 35]. The absence of *scpB* and *bac* in bovine isolates has also been reported by other authors [2, 32, 33, 36]. In the study by Pang et al. [12], in which they compared GBS from human, bovine, fish and environment, the bovine strains were the only ones that did not harbour *scpB* and *lmb*. On the other hand, other authors detected *scpB* in 98%–100% of human isolates and only in 33%–44% of bovine ones [19, 37]. Regarding *bac*, our results are similar to those of Corrêa et al. [2] and Dmitriev et al. [37], who did not detected it in bovine strains but differ from those of Morach et al. [36] and Maeda et al. [35], who detected *bac* in both origins. GBS hypervirulent adhesin (HvgA) is an ST17‐specific surface‐anchored protein, which was described by Lamy et al. [38] for the detection of ST17 strains. We detected *hvgA* only in human isolates as expected since there are no data on their presence among bovine isolates.

The distribution of *pili* islands appears to determine the capacity for colonisation and a strong correlation of it with origin have been demonstrated [5, 12, 13, 39]. PI‐1/PI‐2b combination has been linked to hypervirulent clones from humans [6, 40], while PI‐2b has been associated with bovine isolates [12, 39, 41]. Pilus typing showed that PI‐1 and PI‐2a were only present in human isolates, while PI‐2b was in both origins. These data are in agreement with those reported by Santos Silva Alvim et al. [13], who detected PI‐2a (with or without PI‐1) as the most common variant among human isolates and PI‐2b (with or without PI‐1) among bovine isolates, while Carra et al. [9] reported that the PI‐1/PI‐2b profile was the most common among bovine isolates and, PI‐1/PI‐2a, among human ones. In our analysis, all bovine isolates showed the PI‐2b profile, whereas among human isolates, six profiles were detected. Similar to Brochet et al. [6], Margarit et al. [42], Springman et al. [10] and Lyhs et al. [4], a low percentage human isolates harbouring PI‐2B was detected.

There were no shared virulence profiles between isolates from both host species, which is consistent with the differences between bovine and human profiles reported by Emaneini et al. [32] and Duarte et al. [33]. Such a divergence could be justified by the fact that human and bovine organisms represent very different environments and may therefore result in different evolutionary trajectories. However, there is increasing evidence that, within dairy farms, GBS can survive outside the bovine udder, providing evidence of possible transmission to humans through direct or indirect cycles [11, 16, 43, 44]. Several evolutionary studies have shown that host specialisation can be reflected in processes associated with gene loss or inactivation [45, 46]; therefore, this loss could be responsible for not allowing invasion and recolonisation of humans with the same efficiency, suggesting that transmission would be more permissive from humans to cattle [45, 47]. The fact that no shared virulence profiles were found and the difference in the distribution of serotype frequencies in both groups, together with the fact that certain virulence factor encoding genes are found only in human isolates (*bac, lmb, scpB, PI-1* and *PI-2a*) and that, of those shared, some show significant association with bovine isolates (*rib, spb1* and *PI-2b*), would suggest the coexistence of two subpopulations of *S. agalactiae*, each one associated with a different origin.

In order to treat *S. agalactiae* infections, both in humans and cattle, the most commonly used antibiotics are beta‐lactams, macrolides and lincosamides. Beta lactams resistance was not detected among all the studied isolates [22, 23]. The *ermB* and *linB* genes are responsible for encoding cross‐resistance between macrolides and lincosamides. The e*rmB* gene was detected in about 40% in both bovine and human isolates, in agreement with Cobo‐Ángel et al. [21] and Duarte et al. [33], who also did not find significant differences between origin; *linB* was only detected in bovine isolates as Pinto et al. [30]. Resistance to aminoglycosides is important in treatments against mastitis‐producing bovine *S. agalactiae*, since they are used synergistically with beta‐lactams [48]. The *aphA3* gene, one of the genes conferring resistance to aminoglycosides, was significantly associated with bovine isolates. On the other hand, tetracyclines are used to treat other systemic pathologies within the dairy farm. The main mechanisms of tetracycline resistance are active expulsion (efflux pump) and ribosomal protection (binding of a protein to the ribosome), encoded among other genes by *tetM* and *tetO* [49], with *tetO* being the most prevalent in cattle and, *tetM*, in humans [31, 33], which was confirmed by our data. On the contrary, Cobo‐Angel et al. [16] reported that *tetM* was significantly more prevalent among bovine isolates than among humans. Antimicrobial susceptibility data allowed grouping the isolates into nine profiles, five of which were shared by both origins. Significant association was found between *tetO, ermB-tetO*, and S (susceptible to all tested antimicrobial agents) and bovine isolates, and *tetM* and human isolates.

The detection of multiple STs in Argentinean *S. agalactiae* isolates from both human and bovine sources underscores the genetic heterogeneity of circulating strains. Among the singletons reported so far, ST1640 (here informed) is closely related to ST1614, subsequently assigned to another Argentinean bovine isolate, whose origin is unknown [50]. On the other hand, it was not possible to assign ST to isolate B28, since the genome presented an atypical sequence for *glcK,* a gene encoding a glucose kinase with a predominant function in glucose fermentation [27]. In works in which MLST was performed in isolates from tilapia, similar cases were reported in relation to the same genetic sequence [51, 52]. On the other hand, human isolates also showed atypical *glcK* alleles due to an indel event in this locus [53, 54]. In bovine GBS isolates, the atypical *glcK* allele was found across the major CCs identified in the GBS population, CC1, CC61/67, CC91 and CC103, raising questions about the classification of *glcK* as a housekeeping gene in GBS [53].

Figure 6 shows that in the countries taken into account in the studied period, the most STs were exclusively detected in one or other host; however, out of this period, some countries presented strains with the same STs in both human and bovine lineages (data not shown), highlighting the importance of integrating host and geographic information in epidemiological surveillance. In relation to Argentina, it highlights the low overlap of STs between Argentina and other countries, reinforcing the notion of a possible geographic differentiation. Particularly, ST1640 and ST2276 represent novel locally emerging lineages, indicating ongoing microevolution of the cattle‐specific population. In addition to host‐specific patterns, some few STs were reported during this period in different countries and host: ST17, a hypervirulent lineage commonly associated with human pathogenicity, was detected in both Argentina and Brazil, in human and bovine isolates, respectively, ST4, in Bangladesh (human) and Italy (bovine).

Unlike Lyhs et al. [4] and Carra et al. [9] who studied sympatric GBS isolates obtained from human and cattle isolates obtained in Finland and Sweden during 2010–2012 and from the Emilia Romagna region of Italy in 2018, respectively, we found no evidence of common isolates between both origin isolates. However, to reach these results those authors relied exclusively onto serotypes, pilus island typing and MLST. In our work, in addition to the pilus islands, 12 other virulence genes were taken into account. Boonyayatra et al. [55] studying isolates from Thailand (2011 to 2014) detected ST103 in bovine isolates and a human one. In other study, bovine GBS clades detected in a recent work from Brazil have rarely been reported in humans, suggesting limited risk of interspecies transmission of GBS in that country [53]. On the contrary, the distribution of *S. agalactiae* clones reported in herders and dairy cows in Colombia and Denmark has allowed other authors to postulate the lack of host specificity for some lineages (ST1, ST23 and ST130) and the possibility of direct transmission, especially when the two hosts occupy the same biological environment [16, 18]. The heterogeneity in the published results suggests the need for integrated cross‐host surveillance in each region to better understand transmission dynamics of this pathogen.

In *S. agalactiae,* host‐specific environment, mammary gland and human mucosa, and antimicrobial exposure may have influenced lineage distribution and diversification across. The observation of distinct CC and virulence profiles associated with different hosts, especially bovine‐associated genomic traits such as lactose metabolism operon and genes involved in mammary gland survival which enhance survival and growth in milk‐rich environments have been described [53, 56]. On the other hand, differential antimicrobial exposure such as the frequent antimicrobial use in dairy production systems may also contribute to modelling the population structure in *S. agalactiae*.

### 4.1. Limitations

The main limitations of this study included the unequal sample sizes, the fact that MLST was performed on only the 8 isolates which was possible to be sequenced and that the virulence was analysed using PCR‐based profiling (presence or not of genes and not virulence factor expression).

## 5. Conclusions

The relevance of this work lies in the fact that is one of the few studies worldwide that compare cattle and human *S. agalactiae* isolates obtained in the same geographical area and in the same period. The genetic characteristics among the isolates demonstrate differences between the two populations, questioning the GBS zoonotic status. No virulence genetic profiles or shared lineages between bovine and human isolates were detected. Although these data do not provide conclusive evidence on the ability or not of bovine‐associated GBS to cause invasive infections in humans, it can be noted that in this region, in the studied period, there were not GBS circulating from cattle and humans sharing the same genetic characteristics. It does not mean that interspecies transmission of GBS, zoonotic or anthroponotic is not possible, at least under certain conditions. In order to test this hypothesis, isolates obtained in the same dairy farm, from cattle and people in contact with it, should be studied.

## Author Contributions

A. M. Sanso: conception and design​ of the study, supervision, project administration and funding acquisition. L. B. Hernandez and A. M. Sanso wrote the manuscript. L. B. Hernandez, A. Bustamante and J. Cadona performed the molecular characterisation and the statistical analysis; C. Cacciato, G. Gerez and M. Banchiero collaborated with laboratory assays.

## Funding

This work was supported by grants from the fund for the National Agency for Scientific and Technological Promotion, Argentina (PICT 1139‐17), National Council for Scientific and Technical Research‐CONICET‐(PIP 22‐25‐0035) and Williams Foundation.

## Disclosure

All authors read and approved the final manuscript.

## Ethics Statement

The authors have nothing to report.

## Consent

The authors have nothing to report.

## Conflicts of Interest

The authors declare no conflicts of interest.

## Data Availability

Most data generated or analysed during this study are included in this article. Data on genomic analysis are available from the corresponding author upon reasonable request.
